# Human amniotic mesenchymal stem cells inhibit hepatocellular carcinoma in tumour‐bearing mice

**DOI:** 10.1111/jcmm.15668

**Published:** 2020-08-14

**Authors:** Quan‐Wen Liu, Jing‐Yuan Li, Xiang‐Cheng Zhang, Yu Liu, Qian‐Yu Liu, Ling Xiao, Wen‐Jie Zhang, Han‐You Wu, Ke‐Yu Deng, Hong‐Bo Xin

**Affiliations:** ^1^ The National Engineering Research Center for Bioengineering Drugs and the Technologies Institute of Translational Medicine Nanchang University Nanchang China; ^2^ School of Life and Science Nanchang University Nanchang China; ^3^ Department of Obstetrics and Gynecology The First Affiliated Hospital of Nanchang University Nanchang China; ^4^ Department of Respiratory and Critical Care Medicine The First Affiliated Hospital of Nanchang University Nanchang China

**Keywords:** conditional medium, hepatocellular carcinoma, human amniotic mesenchymal stem cells, IGF‐1R/PI3K/AKT signalling, Wnt/β‐catenin signalling

## Abstract

Hepatocellular carcinoma (HCC) is the third leading cause of the cancer‐related death in the world. Human amniotic mesenchymal stem cells (hAMSCs) have been characterized with a pluripotency, low immunogenicity and no tumorigenicity. Especially, the immunosuppressive and anti‐inflammatory effects of hAMSCs make them suitable for treating HCC. Here, we reported that hAMSCs administrated by intravenous injection significantly inhibited HCC through suppressing cell proliferation and inducing cell apoptosis in tumour‐bearing mice with Hepg2 cells. Cell tracking experiments with GFP‐labelled hAMSCs showed that the stem cells possessed the ability of migrating to the tumorigenic sites for suppressing tumour growth. Importantly, both hAMSCs and the conditional media (hAMSC‐CM) have the similar antitumour effects in vitro, suggesting that hAMSCs‐derived cytokines might be involved in their antitumour effects. Antibody array assay showed that hAMSCs highly expressed dickkopf‐3 (DKK‐3), dickkopf‐1 (DKK‐1) and insulin‐like growth factor‐binding protein 3 (IGFBP‐3). Furthermore, the antitumour effects of hAMSCs were further confirmed by applications of the antibodies or the specific siRNAs of DKK‐3, DKK‐1 and IGFBP‐3 in vitro. Mechanically, hAMSCs‐derived DKK‐3, DKK‐1 and IGFBP‐3 markedly inhibited cell proliferation and promoted apoptosis of Hepg2 cells through suppressing the Wnt/β‐catenin signalling pathway and IGF‐1R‐mediated PI3K/AKT signalling pathway, respectively. Taken together, our study demonstrated that hAMSCs possess significant antitumour effects in vivo and in vitro and might provide a novel strategy for HCC treatment clinically.

## 1 INTRODUCTION

Hepatocellular carcinoma (HCC), which accounts for 80%‐90% of primary liver cancer, is the third leading cause of cancer‐related death worldwide.^1^ In recent years, although the level of screening, diagnosis and treatment of liver cancer has increased, the incidence and mortality of liver cancer have been increasing.^2^ Furthermore, intrahepatic metastasis of primary liver cancer can lead to tumour recurrence, making the treatment of liver cancer very difficult. Therefore, it is urgent to develop new methods to control the growth and metastasis of liver cancer clinically.

Mesenchymal stem cells (MSCs) are a multi‐potential cell that can differentiate into osteocytes, chondrocytes and adipocytes. MSCs also have the abilities of self‐renew and have low tumorigenicity.^3^ MSCs exhibit relatively low immunogenicity and do not stimulate lymphocyte proliferation, thus avoiding immune rejection.^4^ These characteristics of MSCs make them very suitable for cell therapy. The most attractive feature of MSCs is that it can specifically migrate to the tumour site, which indicates its application in tumour therapy. To control tumour growth, MSCs can be genetically engineered to express therapeutic genes,^5^, ^6^, ^7^ loaded with drugs^8^ or be infected with oncolytic viruses.^9^, ^10^ Numerous studies shown that MSCs can directly and/or indirectly affect the development of multiple types of cancer.^11^, ^12^ They are recruited to tumorigenic sites and secrete factors, which have been proved to have antitumour and/or protumour effects.^13^ MSC‐conditioned medium (CM) contains a variety of secretion factors and growth factors, which can promote or inhibit the proliferation, migration or apoptosis of tumour cells and play an important role in regulating the occurrence and development of tumours. Due to different secreted factors, MSCs and MSC‐CM from different sources often have different or even opposite effects in the development of tumours. Emerging evidence demonstrates that MSCs and MSC‐CM have been efficiently applied to treat various types of cancers.^14^, ^15^ In recent years, human amniotic mesenchymal stem cells (hAMSCs) have been considered to be the stem cell with the most application prospect clinically. However, it has not been fully understood how hAMSCs affect HCC cell properties in vivo and in vitro, for example proliferation, apoptosis, migration and invasion.

Recently, we have systemically investigated the morphology, phenotype, pluripotency, tumorigenicity and growth potency of hAMSCs.^4^ Here, we established Hepg2 or Hepg2/hAMSCs co‐injected xenografted BALB/c nude mouse models, and therapeutic effects of hAMSCs in Hepg2 xenografted model mouse were evaluated. We observed that hAMSCs or hAMSCs‐CM had the similar antitumour effects in vivo and in vitro by inducing cell cycle arrest and promoting cell apoptosis, suggesting that the secreted cytokines from hAMSCs might be involved in their antitumour effects. Antibody array assay showed that hAMSCs highly expressed DKK‐3, DKK‐1 and IGFBP‐3, which inhibited liver cancer growth through suppressing Wnt/β‐catenin and IGF‐1R signalling pathways. Taken together, we demonstrated that hAMSCs‐derived trophic factors possess anti‐HCC effect in vitro and in vivo, suggesting that hAMSCs may provide a novel therapeutic strategy for the treatment of liver cancer.

## 2 MATERIALS AND METHODS

### 2.1 Isolation, culture and expansion of hAMSC_S_

After obtaining the oral consent of the donor, fresh amniotic membrane tissue was collected from the department of the obstetrics and gynaecology, the first affiliated hospital, Nanchang University. hAMSCs were prepared as previously described.^4^, ^16^

### 2.2 Lentiviral infection

The lentiviral GFP expression vector pHBLV‐IRES‐ZsGreen‐PGK‐puro was purchased from Hanbio (Shanghai, China). hAMSCs at passage 2‐3 were infected with virus supernatant (multiplicity of infection 60) containing polyamine (6 mg/mL) for 24 hours. Subsequently, puromycin (3 mg/mL; Sigma‐Aldrich) was used to select GFP‐positive cells. The percentage of GFP‐labelled hAMSCs was determined by immunofluorescence every day. Once the percentage of GFP‐labelled hAMSCs was higher than 95%, puromycin was removed from the culture medium.

### 2.3 Flow cytometry analysis, adipogenic and osteogenic differentiation

The surface molecular markers and differentiation potential of cultured hAMSCs and GFP‐labelled hAMSCs were determined by flow cytometry analysis and adipogenic and osteogenic differentiation as previously described.^4^ The antibodies used for flow cytometry analysis are listed in Table S1.

### 2.4 Animal models

8‐week‐old NOD‐SCID male mice and BALB/c nude mice were obtained from Changsha SLAC Laboratory Animal Company (Changsha, China, http://www.hnsja.com/). All animal experiments were performed according to institutional guidelines and approved by the Animal Care and Use Committee of Nanchang University.

### 2.5 Hepg2 cell xenograft model and fluorescent imaging in vivo

The human hepatocarcinoma cell line Hepg2 was obtained from ATCC (Manassas, VA) and maintained in H‐DMEM (Thermo Fisher) containing 100 U/mL penicillin, 100 μg/mL streptomycin and 10% FBS. Cells were incubated in a 5% CO_2_‐humidified incubator at 37°C. Hepg2 cells (5 × 10^6^) were injected subcutaneously into the dorsal region of BALB/c nude mice. 1.5 × 10^6^ GFP‐labelled hAMSCs in 300 μL 1 × PBS or 300 μL 1 × PBS alone as a control was intravenously injected after 6, 12 and 18 days of Hepg2 transplantation. The longest size (a) and the shortest size of (b) tumour were measured with vernier calliper (Mitutoyo Co., Tokyo, Japan) every day for 24 days. The tumour volume calculation formula is V= (1/2)ab^2^. Eighteen days after cell injection, the mice were anaesthetized first, and the mice were visualized with whole‐body fluorescent imaging system (LB983; Berthold, Germany). Then, the mice were killed, and the tissues of tumour, heart, liver, brain, kidney, spleen, lung and pancreas were isolated and visualized with whole‐body fluorescent imaging system.

### 2.6 Histopathology and TUNEL assay

Tumour tissues were processed for paraffin by slicing into 5‐μm‐thick section. Immunohistochemistry was performed on tumour sections to detect PCNA (1:1000, mouse monoclonal antibody, Abcam, Nanchang, China). Apoptosis assay was performed on tumour tissue sections by TUNEL assay kit (Millipore, USA) according to the manufacturer's instruction.

### 2.7 Production of CM and experiment of co‐culture in vitro

hAMSC‐CM was prepared as previously described.^4^ Briefly, hAMSCs were grown in normal culture medium, once the cells reached to 80% confluency, the medium was changed with H‐DMEM containing 100 U/mL penicillin and 100 μg/mL streptomycin. CM was collected after 48 hours and centrifuged at 1500 rpm for 5 minutes to ensure complete removal of cellular debris. CM was then concentrated 10‐fold by using an Amicon® Ultra 3K device (Millipore, Sigma, USA).

The in vitro experiment was divided into three groups. Control group: 1.5 × 10^5^ Hepg2 cells were cultured in 3 mL of H‐DMEM containing 10% FBS, 100 U/mL penicillin and 100 μg/mL streptomycin in 6‐well dish. hAMSC group: a co‐culture transwell chamber (2.4 cm diameter, 0.4 μm pore size; Corning) was used to evaluate the effect of hAMSCs on proliferation and apoptosis of Hepg2 cells in vitro. Hepg2 cells were seeded into lower chamber with 2 mL of H‐DMEM containing 10% FBS, 100 U/mL penicillin and 100 μg/mL streptomycin. 1.5 × 10^5^ hAMSCs were resuspended in 1 mL of the same medium and seeded in upper compartment. hAMSC‐CM group: Hepg2 cells were cultured with 3 mL H‐DMEM supplemented with 10% hAMSC‐CM (10×), 10% FBS, 100 U/mL penicillin and 100 μg/mL streptomycin in 6‐well dish. Samples were collected after culture of 24 and 48 hours, respectively.

To neutralize DKK‐1, DKK‐3 and IGFBP‐3, hAMSC‐CM was incubated with rabbit anti‐DKK‐1(1:100, mouse monoclonal antibody, Santa Cruz, Nanchang, China), anti‐DKK‐3 (1:70, rabbit monoclonal antibody, Abcam) and anti‐IGFBP‐3 (1:200, rabbit monoclonal antibody, CST, Nanchang, China) antibodies at 4^◦^C for 24 hours and subsequently used to treat Hepg2 cells for 48 hours. The normal rabbit IgG was used as negative control.

### 2.8 Tumour cell proliferation analysis

Cell proliferation of different groups was determined using a CCK‐8 kit (Dojindo Laboratories, Kumamoto, Japan), according to the manufacturer's instructions from 24 to 48 hours after cells were plated. Absorbance values were measured at a wavelength of 450 nm using a microplate spectrophotometer (Bio‐Rad).

### 2.9 Western blot analysis and immunofluorescence

Western blot analysis and immunofluorescence were preformed as previously described^4^; the primary antibodies are listed in Table S1, including anti‐GAPDH (1:10 000, rabbit monoclonal, Abcam), anti‐PCNA (1:1000, mouse monoclonal, Abcam), anti‐Ki67 (1:1000, mouse monoclonal, Abcam), anti‐cyclin E1 (1:1000, rabbit monoclonal, Abcam), anti‐cyclin D1 (1:10 000, rabbit monoclonal, Abcam), anti‐cyclin A2 (1:2000, rabbit monoclonal, Abcam), anti‐cyclin B1 (1:2000, rabbit monoclonal, Abcam), anti‐cleaved‐PARP (1:10 000, rabbit monoclonal, Abcam), anti‐cleaved‐caspase‐3 (1:1000, rabbit polyclonal, CST), anti‐IGFBP3 (1:1000, rabbit monoclonal, CST), anti‐DKK‐1(1:1000, mouse monoclonal, Santa Cruz), anti‐DKK‐3 (1:1000, rabbit monoclonal, Abcam), anti‐P‐Gsk3β (1:1000, rabbit polyclonal, Abcam), anti‐Gsk3β (1:1000, mouse monoclonal, Abcam), anti‐β‐catenin (1:5000, rabbit monoclonal, Abcam), anti‐AKT (1:1000, mouse monoclonal, Abcam), anti‐P‐AKT (1:1000, mouse monoclonal, Abcam), anti‐PI3K (1:1000, rabbit polyclonal, Abcam), P‐PI3K (1:1000, rabbit polyclonal, CST), anti‐IGF‐1R (1:1000, rabbit monoclonal, CST), anti‐P‐IGF‐1R (1:1000, rabbit monoclonal, CST), anti‐N‐cadherin (1:5000, rabbit monoclonal, Abcam), anti‐Bcl‐2 (1:1000, mouse monoclonal, Abcam) and anti‐Bax (1:1000, mouse monoclonal, Abcam).

### 2.10 Antibody protein array

Human cytokine arrays (QAH‐CAA‐440‐1, RayBiotech, Norcross, USA) were used to measure the expression of 440 cytokines in serum‐free culture supernatants at 48 hours from hAMSCs, according to the manufacturer's instructions.

### 2.11 Small interfering RAN (siRNA)‐mediated silencing of DKK‐3, DKK‐1 and IGFBP‐3 expression

The siRNA targeting DKK‐3, DKK‐1 and IGFBP‐3 were purchased from Ribo‐Bio (Guangzhou, China). hAMSCs were transfected with siDKK‐3, siDKK‐1 or siIGFBP‐3 at a final concentration of 50 nM using ribo*FECT* ^TM^ CP Reagent (RayBiotech, Guangzhou, China) according to the manufacture's instructions. hAMSCs transfected with only transfection reagent (without siRNA) (hAMSC‐siMOCK) were used as controls.

### 2.12 Statistical analysis

All data are presented as mean ± standard deviation (SD). Data were analysed by Student's *t* test or one‐way analysis of variance (ANOVA). Differences between values were considered significant at *P* < .05.

## 3 RESULTS

### 3.1 Identification and characterization of hAMSCs and GFP‐labelled hAMSCs

**FIGURE 1:**
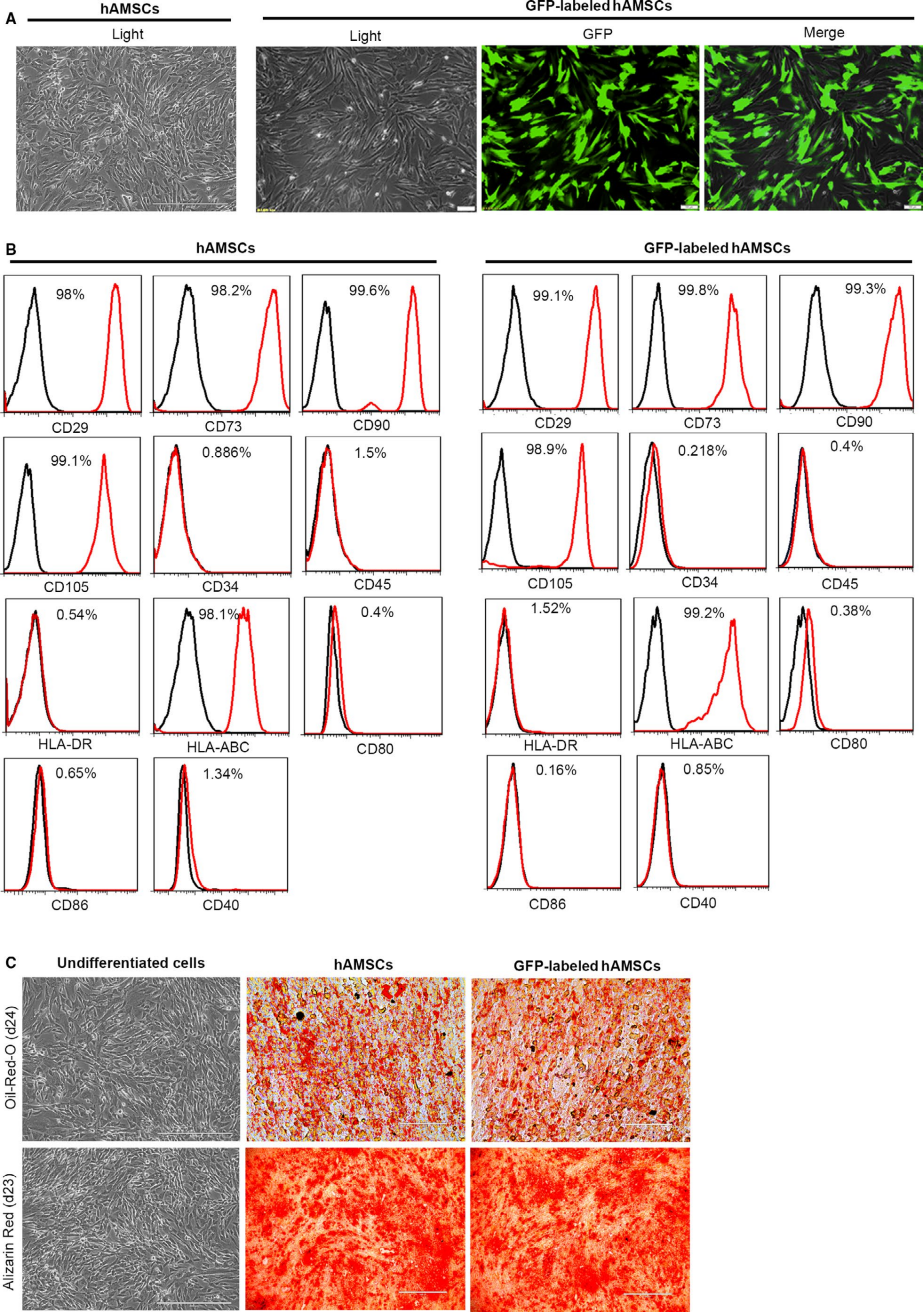
Characterization of cell morphology and markers of hAMSCs and GFP‐labelled hAMSCs. A, Representative images of cultured hAMSCs and GFP‐labelled hAMSCs. B, Detection of surface markers in hAMSCs, GFP‐labelled hAMSCs (red) and in isotype controls (black) by flow cytometry. hAMSCs and GFP‐labelled hAMSCs were positive for CD29, CD90, CD73, CD105, HLA‐ABC, but negative for CD34, CD45, HLA‐DR, CD80, CD86 and CD40. C, Adipogenic differentiation of hAMSCs and GFP‐labelled hAMSCs was demonstrated by staining with oil red O, and osteogenic differentiation was demonstrated by Alizarin Red staining

The GFP‐labelled hAMSCs (GFP‐hAMSCs) were prepared by lentiviral infection for cell tracking. As shown in Figure 1A, more than 95% of infected hAMSCs were GFP‐positive after puromycin selection. Compared with hAMSCs, the morphology of GFP‐hAMSCs did not change significantly; it was spindle‐shaped and fibroblast‐like and grew in adherent monolayer. In the medium containing bFGF, hAMSCs and GFP‐hAMSCs proliferated rapidly with an average doubling time of two days (Figure 1A). Flow cytometry showed that both hAMSCs and GFP‐hAMSCs expressed MSCs marker proteins CD105, CD73, CD90, CD29 and HLA‐ABC, a major histocompatibility protein, but did not express CD34 and CD45, the hematopoietic stem cell marker proteins. hAMSCs and GFP‐hAMSCs also negative for major histocompatibility proteins HLA‐DR and HLA‐ABC co‐stimulate molecules CD80, CD86 and CD40 (Figure 1B). In vitro, both hAMSCs and GFP‐hAMSCs can be induced to differentiate into osteoblasts and adipocytes under osteogenic and adipogenic differentiation conditions (Figure 1C). The above results show that hAMSCs and GFP‐hAMSCs both express specific molecular markers of MSCs and have low immunogenicity and multi‐differentiation potential, the transfection of GFP does not affect the characteristics and proliferation ability of hAMSCs. In addition, our previous research results show that hAMSCs had no tumorigenicity in vitro and in vivo. All these advantages make hAMSCs and GFP‐hAMSCs have great clinical application potential.

### 3.2 hAMSCs inhibit tumour growth in vivo

**FIGURE 2:**
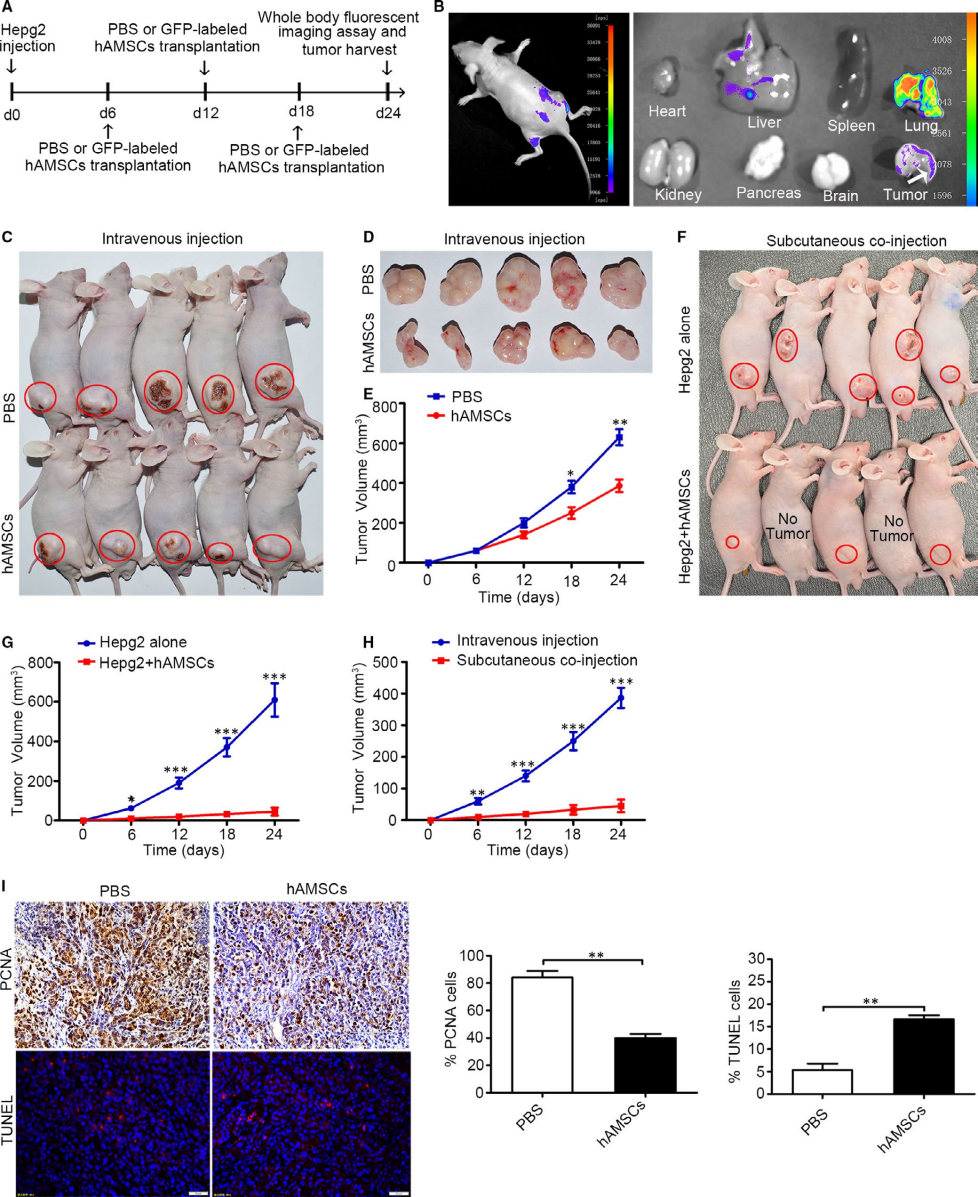
hAMSCs exhibit tumour‐homing and tumour‐suppressive properties in HCC mouse model. A, Experimental schematic of Hepg2‐induced hepatocellular carcinoma in the BALB/c nude mice model. The experiments were conducted in two groups including the PBS group and GFP‐labelled hAMSC group. B, Monitoring of hAMSCs tracking to tumour lesions in HCC mouse model on day 24 after Hepg2 transplantation by whole‐body fluorescent imaging assay. The results showed that GFP‐positive cells were detected around tumour site and in the tumour tissues. C and D, Gross observation of subcutaneous xenografts of Hepg2/PBS and Hepg2/hAMSCs intravenous‐injected nude mice. The red circles represent the tumour location. E, The average volume of hAMSC‐injected tumours was significantly smaller than that of PBS‐injected tumours at day 18 and day 24 (n = 5). F, Gross observation of subcutaneous xenografts of Hepg2 alone and Hepg2/hAMSCs co‐injected nude mice. The red circles represent the tumour location. G, The average volume of Hepg2/hAMSCs subcutaneous co‐injected tumours was significantly smaller than that of Hepg2 alone injected tumours at day 6, 12, 18 and 24 (n = 5). H, The average volume of Hepg2/hAMSCs subcutaneous co‐injected tumours was significantly smaller than that of hAMSCs intravenous‐injected tumours at days 6, 12, 18 and 24. I, Proliferation and apopsis of Hepg2 cells were tested by immunohistochemistry using antibodies against PCNA and TUNEL staining in PBS and hAMSCs intravenous‐injected tumour tissues. Results are shown as mean ± SD

A mouse tumour model was generated by injecting Hepg2 cells into the dorsal region of BALB/c nude mice. After 6 days of the initial injection of Hepg2 cells, the xenograft tumours were reached to a volume of ~ 60 mm^3^. The GFP‐hAMSCs (1.5 × 10^6^ cells in 300 μL 1 × PBS) or PBS (300 μL) was intravenously injected at day 6, day 12 and day 18 after Hepg2 cell inoculation, and the tumour sizes were measured every day for 24 days (Figure 2A). The results from the whole‐body fluorescent imaging showed that hAMSCs were migrated to the tumorigenic site at day 24 (Figure 2B). As shown in Figure 2C‐E, the tumour volumes were significantly reduced in hAMSC group (mean volume, 386.67 ± 44.97 mm^3^) compared with PBS group (mean volume, 630.84 ± 57.15 mm^3^) at day 24 after the tumour introduction, and the mean size of the tumours in hAMSC group was reduced by ~ 39% after administration of hAMSCs for 18 days compared with control mice. To further confirm the inhibitory effect of hAMSCs on Hepg2 cells in vivo, we then injected Hepg2 cells alone (5 × 10^6^; n = 5) or cell mixture of Hepg2 cells (5 × 10^6^) and hAMSCs (5 × 10^6^) (n = 5) into BALB/c nude mice. We observed that there was no tumour or very small tumour formation in the mice co‐injected with Hepg2 plus hAMSCs (mean volume, 45 ± 40 mm^3^) over a time period of 24 days compared with the animals implanted with Hepg2 cells alone (mean volume, 609 ± 168.76 mm^3^) (Figure 2 F‐G). In addition, the tumour volumes were significantly reduced in Hepg2/hAMSCs co‐injection group compared with hAMSC intravenous injection group at days 6, 12, 18 and 24 after the tumour introduction (Figure 2H). TUNEL assay and immunohistochemistry assay showed that intravenous‐injected hAMSCs significantly increased TUNEL‐positive cells and decreased PCNA‐positive cells in tumour tissues compared with the PBS groups (Figure 2I). These results indicated that the hAMSCs injected intravenously were able to efficiently home to the tumorigenic sites and in turn markedly inhibited the tumours growth through suppressing proliferation and promoting apoptosis of the tumour cells.

### 3.3 hAMSCs or hAMSC‐CM inhibited proliferation and promoted apoptosis of Hepg2 cells via a paracrine manner in vitro

**FIGURE 3:**
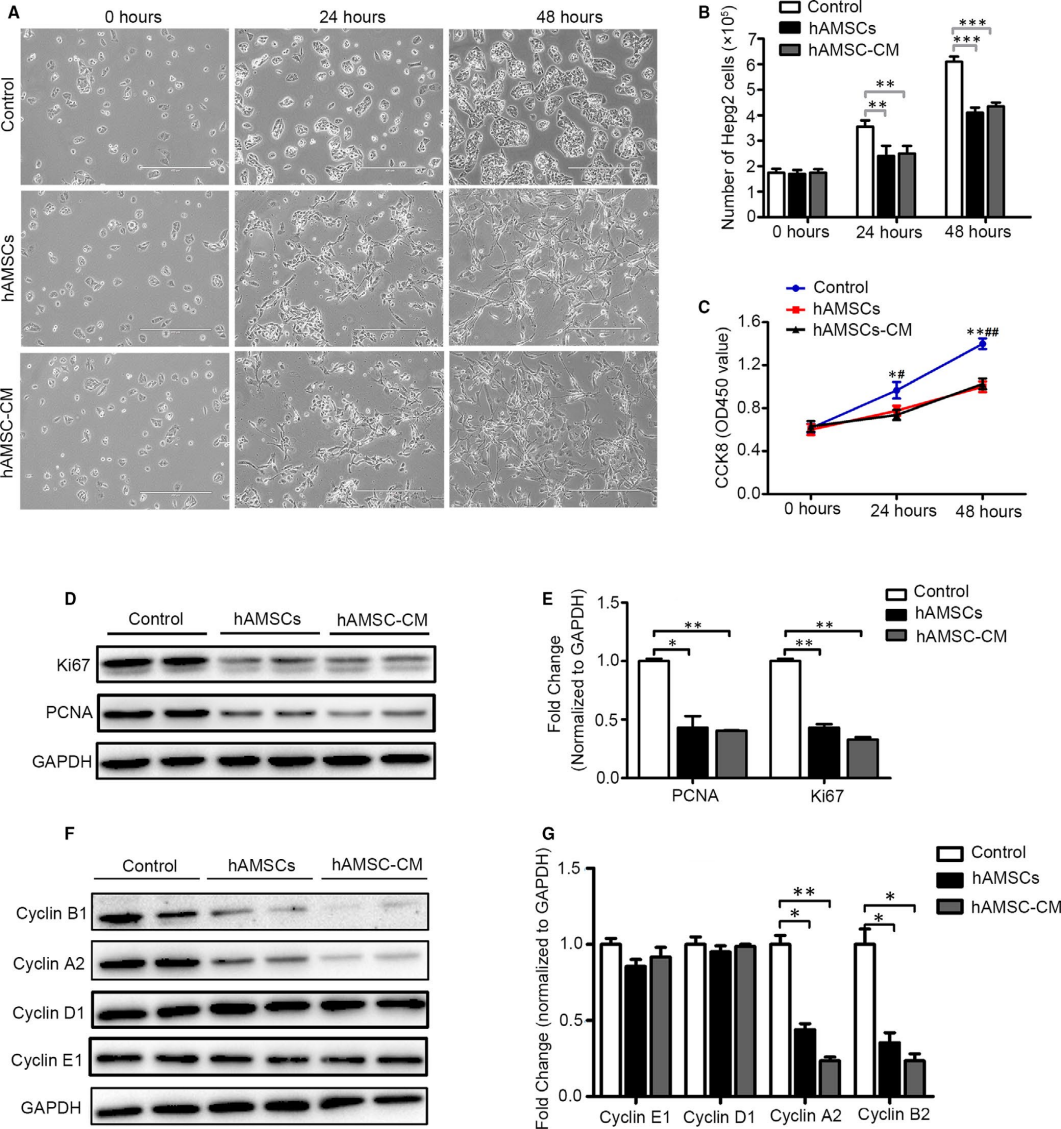
Effect of hAMSCs and hAMSC‐CM on cell proliferation in Hepg2 cells. A, Representative images of Hepg2 cells observed under a light microscope after cultured with normal medium (control), hAMSCs or hAMSC‐CM for 0, 24 and 48 hours. B, Number of Hepg2 cells at 0, 24 and 48 hours post‐treated of normal medium, hAMSCs and hAMSC‐CM were tested by cell counting assay. C, CCK‐8 assay for cell proliferation of Hepg2 cells in the control, hAMSCs and hAMSC‐CM group at different time‐points. D, Western blot analysis of Ki67 and PCNA in Hepg2 cells treated with normal medium, hAMSCs and hAMSC‐CM for 48 h. E, Quantitative analyses of PCNA and Ki67 protein levels in Hepg2 cells as shown in (D). F, Expression of cyclin B1, cyclin A2, cyclin D1, cyclin E1 examined by Western blot in Hepg2 cells in different groups. G, Quantitative analyses for relative protein level of Hepg2 cells as shown in (F). Results are shown as mean ± SD

Next, to determine whether the antitumour effects were directly mediated by hAMSCs, the secreted cytokines from hAMSCs (hAMSCs‐CM) were used for evaluating the antitumour effects with HCC cell lines in vitro, in which Hepg2 cells were co‐cultured in 1:1 ratio with hAMSCs or treated with 10% hAMSC‐CM (10 ×). The results showed that Hepg2 cells lost their cobblestone‐like morphology and became thinner and longer in both hAMSCs and hAMSC‐CM groups after 24 and 48 hours of treatment (Figure 3A). The numbers of Hepg2 cells were sharply decreased in hAMSC and hAMSC‐CM groups compared with the control group (Figure 3B). CCK8 assay further confirmed that the Hepg2 cells were significantly inhibited by hAMSCs and hAMSC‐CM compared to the control at 24 and 48 hours; however, there was no obvious difference between the hAMSC group and hAMSC‐CM group (Figure 3C). Western blot analysis revealed that the expressions of Ki67 and PCNA (two proliferation‐related proteins) of Hepg2 cells were significantly decreased in hAMSC and hAMSC‐CM groups compared with control group (Figure 3D). Compared to control group, hAMSCs co‐culture and hAMSC‐CM treatment decreased the expression of PCNA by 55.5 ± 8.5% and 59.5 ± 6.3%, respectively, as well as 57 ± 3% and 67 ± 2% for Ki67 (Figure 3E). Western blot analysis also showed that the expressions of cyclin B1 and cyclin A2 were significantly decreased in Hepg2 cells in both hAMSCs and hAMSC‐CM groups compared with control group. By contrast, there were no significant differences for the expressions of cyclin D1 and cyclin E1 in HEPG2 cells between control, hAMSCs and hAMSC‐CM (Figure 3F,G). The above results showed that hAMSCs and hAMSCs‐CM inhibited the proliferation of Hepg2 by the induction of cell cycle arrest at the S and G2 phase.

**FIGURE 4:**
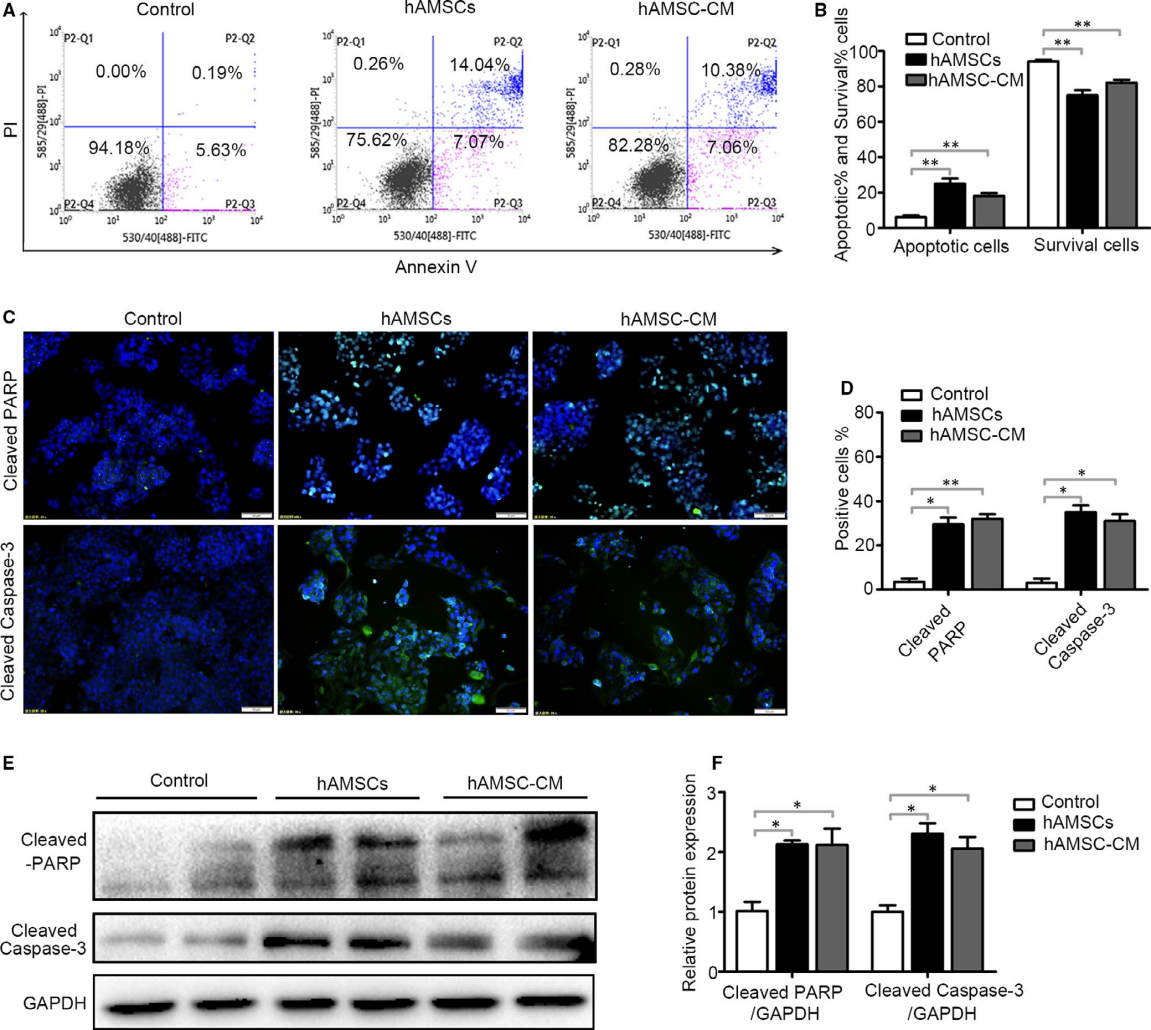
Effect of hAMSCs and hAMSC‐CM on cell apoptosis in Hepg2 cells. A, Hepg2 cells were treated with normal medium (control), hAMSCs and hAMSC‐CM. The apoptosis of cells was assessed by FACS after 48 h of treatment. B, Quantitative analysis of the percentage of apoptotic cells and living cells as shown in A (n = 3). C, Effect of hAMSCs and hAMSC‐CM on cleaved‐PARP and cleaved caspase‐3 was detected in immunofluorescence assay. D, Quantitative analysis of the percentage of cleaved‐PARP positive and cleaved caspase‐3‐positive cells as shown in (C). E, The expression levels of cleaved‐PARP and cleaved caspase‐3 in Hepg2 cells of different groups were analysed by immunoblotting assay. F, Quantitative analysis of the expression of cleaved‐PARP and cleaved caspase‐3 in Hepg2 cells as in (E). Results are shown as mean ± SD

After 48 hours of treatment, we evaluate the influence of hAMSCs and hAMSC‐CM on the apoptosis of Hepg2 cells by flow cytometry analysis. We found that when compared with the control group, hAMSCs and hAMSC‐CM treatment significantly increased the apoptosis rates of Hepg2 cells (Figure 4A). The apoptotic rates of these Hepg2 cells were 6 ± 1.63% for control, 25 ± 4.08% for hAMSCs and 18 ± 2.45% for hAMSCs‐CM, respectively (Figure 4B). Additionally, the immunofluorescence assay and Western blotting analysis showed that both cleaved‐PARP and cleaved caspase‐3 levels in Hepg2 cells were increased in hAMSC and hAMSC‐CM groups compared with control group (Figure 4C–F). These data indicated that hAMSCs and hAMSC‐CM promote apoptosis in Hepg2 cells. Taken together, these results demonstrated that the antitumour effects of hAMSCs were dependent on inhibiting cell proliferation and promoting cellular apoptosis in a paracrine manner.

### 3.4 DKK‐3 and DKK‐1 derived from hAMSCs inhibited Hepg2 cell proliferation through blocking canonical Wnt/β‐catenin signalling pathway

**FIGURE 5:**
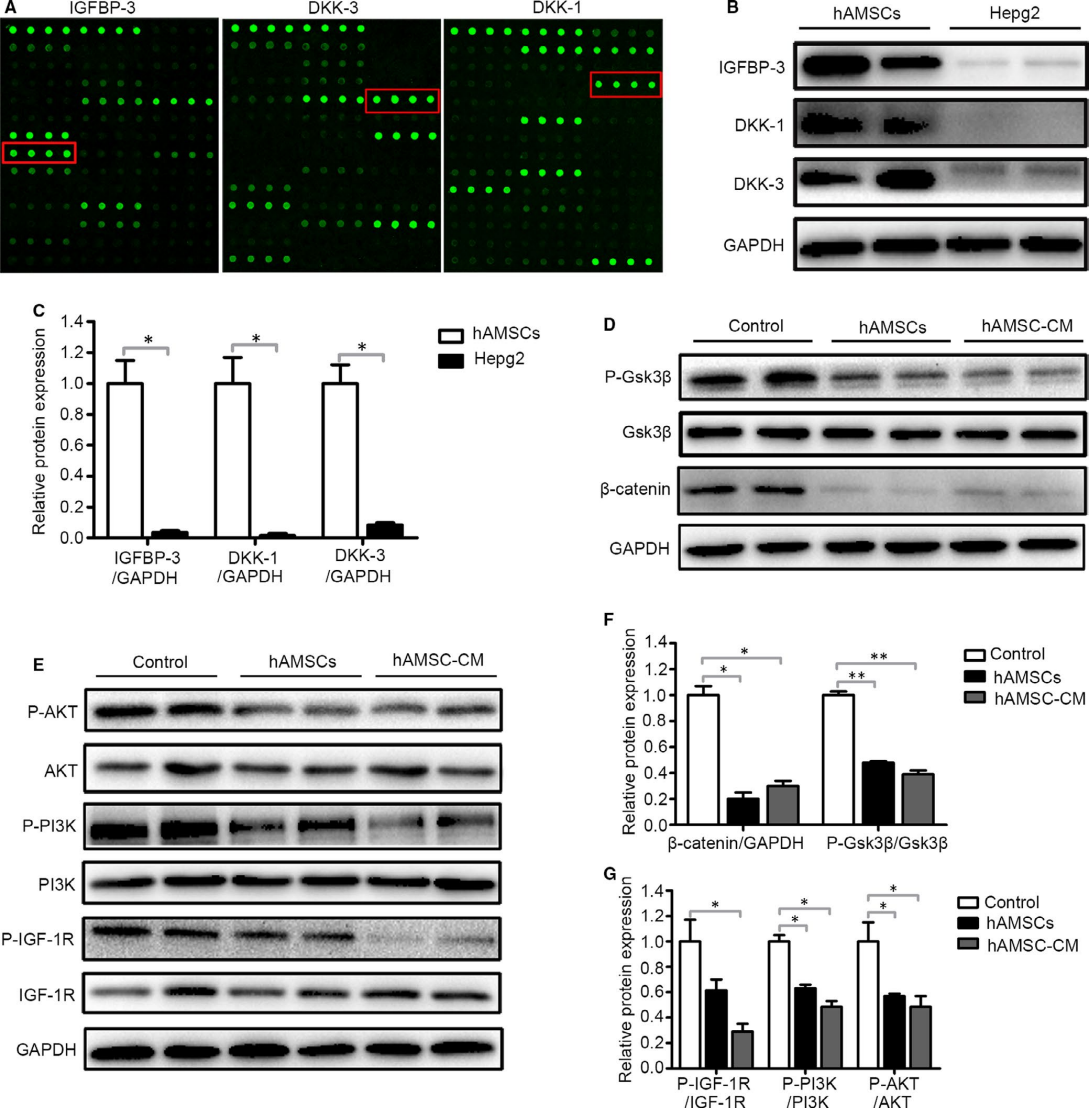
hAMSC‐derived DKK‐3, DKK‐1 and IGFBP‐3 reduced activation of Wnt/β‐catenin and IGF‐1R/PI3K/AKT signalling pathway of Hepg2 cells. A, Representative array images are shown (n = 4). DKK‐3, DKK‐1 and IGFBP‐3 are highlighted with red boxes. B, Western blot analysis of DKK‐3, DKK‐1 and IGFBP‐3 protein levels in hAMSCs and Hepg2 cells. C, Quantitative analysis of the expression of IGFBP‐3, DKK‐1 and DKK‐3 in hAMSCs and Hepg2 cells as in (B). D, Hepg2 cells were treated with normal medium (control), hAMSCs and hAMSC‐CM. Western blot analysis of protein levels of β‐catenin, GSK3β and P‐GSK3β in Hepg2 cells of each treatment group. E, Western blot analysis of protein levels of IGF‐1R, P‐ IGF‐1R, PI3K, P‐ PI3K, AKT and P‐AKT in Hepg2 cells of control, hAMSCs and hAMSC‐CM group. F, Quantitative analysis of the expression of β‐catenin and P‐GSK3β in Hepg2 cells of different groups as in (D). G, Quantitative analysis of the expression of P‐ IGF‐1R, P‐PI3K and P‐AKT in Hepg2 cells of different groups as in (E)

The hAMSCs and hAMSC‐CM have a similar antitumour effect, suggesting that the secreted cytokines from hAMSCs were responsible for the antitumour effect. To elucidate the possible mechanism of the antitumour effects of hAMSCs, the antibody array assay was performed to examine the cytokine levels in the supernatants of hAMSCs. Among 440 cytokines evaluated, the 20 factors with the highest expression amount are shown in Table 1. Interestingly, we found that IGFBP‐3, DKK‐3 and DKK‐1 were highly secreted by hAMSCs; the concentrations were 45 401.20 pg/mL, 37 200.47 pg/mL and 11 930.42 pg/mL, respectively (Figure 5A and Table 1). The concentrations of around 200 cytokines were more than 20 pg/mL and the detail information is shown in Table S2. DKK‐3 and DKK‐1 are Wnt antagonists, which can inhibit the Wnt/β‐catenin signalling pathway. IGFBP‐3 is a member of the family of six insulin‐like growth factor‐binding proteins (IGFBPs) with the most abundant IGFBP in human serum, which controls the bioavailability and bioactivity of IGF. IGF‐1R has the ability to activation of PI3K/AKT pathway by triggering receptor and intracellular targets phosphorylation.^17^ Both Wnt/β‐catenin and IGF signalling pathways play important roles in HCC tumorigenesis.^18^, ^19^ These results indicated that hAMSCs inhibit the proliferation and promote the apoptosis of Hepg2 cells via inhibiting the Wnt/β‐catenin and IGF signalling pathways. Consistent with the finding in antibody arrays, the Western blot analysis further confirmed that DKK‐3, DKK‐1 and IGFBP‐3 were highly expressed in hAMSCs, but not in Hepg2 cells (Figure 5B,C). In Hepg2 cells treated with hAMSCs and hAMSC‐CM, the accumulation of β‐catenin and the expression of P‐GSK3β (S9) were decreased compared with the controls (Figure 5C,F). Furthermore, the expressions of P‐IGF‐1R, P‐PI3K and P‐AKT were down‐regulated in the hAMSCs and hAMSC‐CM‐treated Hepg2 cells (Figure 5E,G), suggesting that both Wnt/β‐catenin and IGF signalling pathways were inhibited when the Hepg2 cells treated with hAMSCs or hAMSC‐CM.

**TABLE 1:** Twenty factors with the highest expression amount that are presented in hAMSC‐CM

	Protein ID	Concentration (pg/mL)		Protein ID	Concentration (pg/mL)
1	PAI‐1	47 508.89	2	TSP‐1	46 888.6
3	**IGFBP‐3**	45 401.2	4	VEGF R1	44 504.73
5	**DKK‐3**	37 200.47	6	ANGPTL4	28 309.84
7	G‐CSF	23 869.68	8	Periostin	22 592.24
9	Nidogen‐1	19 445.92	10	Thrombospondin‐2	19 240.46
11	NCAM‐1	16 793.46	12	CHI3L1	12 115.05
13	**DKK‐1**	11 930.42	14	TIMP‐1	11 697.54
15	Albumin	11 391.94	16	TIMP‐2	9685.848
17	GROa	9143.29	18	bIG‐H3	9048.115
19	B2M	8007.654	20	AMICA	7198.752

The boldface items in the table represent the cytokines secreted by hAMSCs that may involve in their anti‐tumor effect.

**FIGURE 6:**
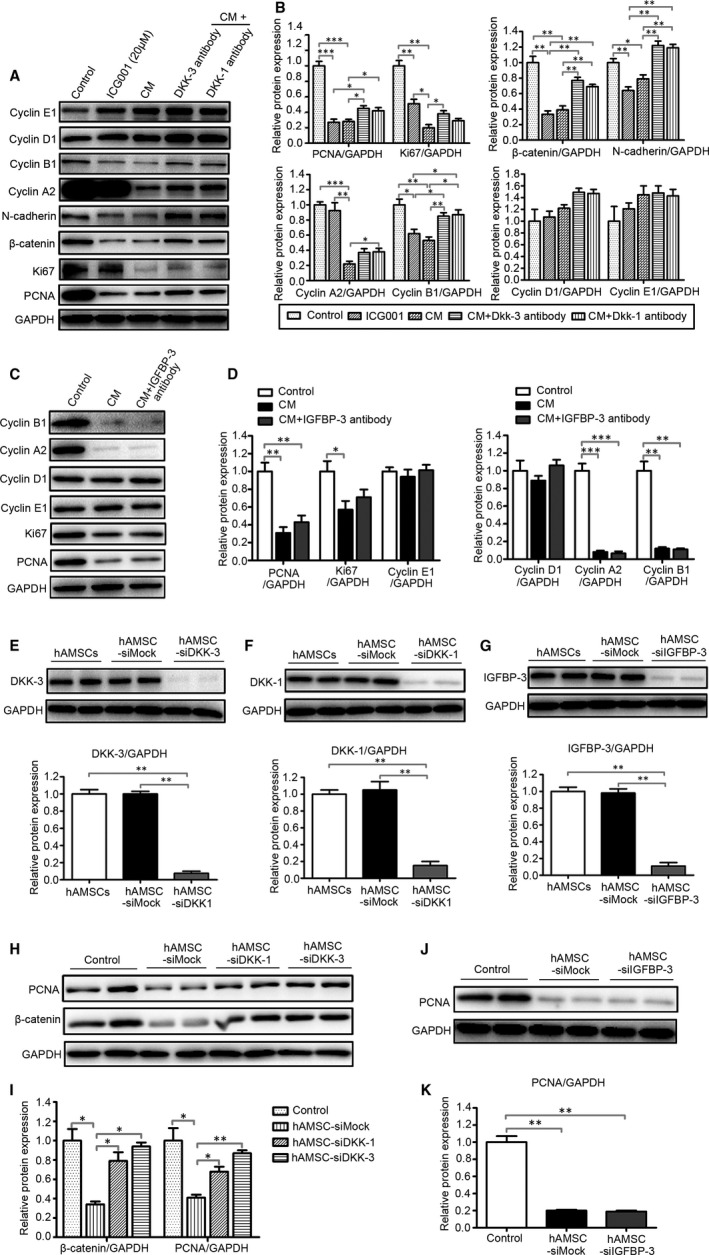
hAMSC‐derived DKK‐3 and DKK‐1 inhibit the proliferation of Hepg2 cells through blocking Wnt/β‐catenin signalling pathway. hAMSC‐CM was pre‐treated with monoclonal antibody against DKK‐1, DKK‐3 and IGFBP‐3 for 24 hours. After which, the Hepg2 cells were treated with normal culture medium, ICG001 (20 μM), CM, CM + DKK‐3 antibody, CM + DKK‐1 antibody and CM + IGFBP‐3 antibody for 48 h. A, The expression of PCNA, Ki67, β‐catenin, N‐cadherin, cyclin A2, cyclin B1, cyclin D1 and cyclin E1 in Hepg2 cells in each group was analysed by Western blot. B, Quantitative analysis of the expression of PCNA, Ki67, β‐catenin, N‐cadherin, cyclin A2, cyclin B1, cyclin D1 and cyclin E1 in Hepg2 cells of different groups as in (A). C, The expression of PCNA, KI67, cyclin A2, cyclin B1, cyclin D1 and cyclin E1 in Hepg2 cells in control (normal medium), CM, CM + IGFBP‐3 antibody group were analysed by Western blot. D, Quantitative analysis of the expression of PCNA, KI67, cyclin A2, cyclin B1, cyclin D1 and cyclin E1 in Hepg2 cells of different groups as in C. The RNAi efficiency of DKK‐3 (E), DKK‐1 (F) and IGFBP‐3 (G) in hAMSCs was assayed by Western blot before co‐culture with Hepg2 cells. H, Western blot analysis showed that the accumulation of β‐catenin and the expression of PCNA were increased in hAMSC‐siDKK3 group and hAMSC‐siDKK1 group when compared with hAMSC‐siMOCK group. I, Quantitative analysis of the expression of PCNA and β‐catenin in Hepg2 cells of different groups as in (H). J, Western blot analysis showed that the expression of PCNA was no significant difference observed in Hepg2 cells co‐cultured with hAMSC‐siMOCK and hAMSC‐siIGFBP‐3. K, Quantitative analysis of the expression of PCNA in Hepg2 cells of different groups as in J. Results are shown as mean ± SD

To further determine the specificity of the hAMSC‐secreted DKK‐3, DKK‐1 and IGFBP‐3 on the inhibition of Hepg2 cells, the antibodies of rabbit anti‐human DKK‐3, anti‐human DKK‐1 and anti‐human IGFBP3 were used in vitro. The results showed that the hAMSC‐CM pre‐treated with the antibodies against DKK‐3 and DKK‐1 partially lost the ability of down‐regulating the expression of β‐catenin and its target protein PCNA, Ki67, N‐cadherin, cyclin A2 and cyclin B1. The expressions of β‐catenin and PCNA in Hepg2 cells were significantly inhibited by β‐catenin inhibitor ICG001 (Figure 6A,B). In contrast, the expressions of PCNA, Ki67, cyclin A2, cyclin B1, cyclin D1 and cyclin E1 in Hepg2 cells were not inhibited after the IGFBP‐3 activity was neutralized by the antibody against IGFBP‐3 (Figure 6C,D). Thus, the data demonstrated that the hAMSC‐CM‐mediated the suppressed cell proliferation in Hepg2 cells was almost abolished by DKK‐1 and DKK‐3 neutralization, indicating that the secreted DKK‐1 and DKK‐3 from hAMSCs contributed to the inhibition of Hepg2 cells. To further confirm the functions of DKK‐3, DKK‐1 and IGFBP‐3, the siRNAs targeting DKK‐3, DKK‐1 and IGFBP‐3 mRNA were used to down‐regulate the expressions of DKK‐3, DKK‐1 and IGFBP‐3 in hAMSCs. As shown in Figure 6E‐G, the expressions of DKK‐3, DKK‐1 and IGFBP‐3 proteins were reduced by 95%, 85% and 91%, respectively, and the DKK‐3 or DKK‐1 siRNA‐transfected hAMSCs partially lost the ability of suppressing the expressions of β‐catenin and PCNA in Hepg2 cells (Figure 6H,I). In contrast, the IGFBP‐3 siRNA‐transfected hAMSCs have the similar ability of decreasing the expression of PCNA in Hepg2 cells compared with hAMSC‐siMOCK (Figure 6J,K). Taken together, these results demonstrated that DKK‐3 and DKK‐1 derived from hAMSCs inhibit Hepg2 cell proliferation through blocking canonical Wnt signalling pathway.

### 3.5 hAMSC‐derived DKK‐3, DKK‐1 and IGFBP3 inhibited apoptosis of Hepg2 cells through blocking Wnt/β‐catenin and IGF‐1R/PI3K/AKT pathways

**FIGURE 7:**
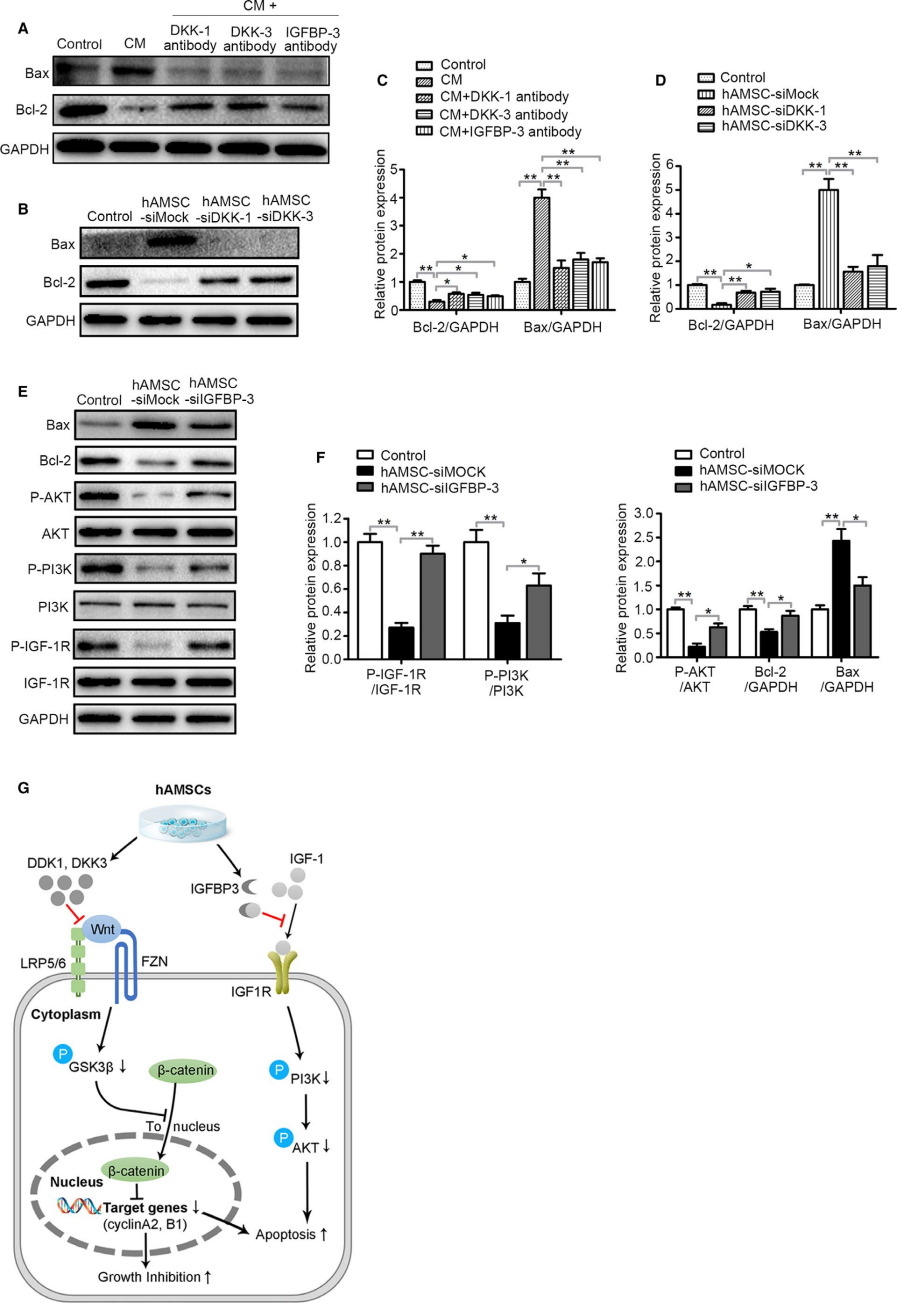
DKK‐3, DKK‐1 and IGFBP‐3 derived from hAMSCs inhibit survival of Hepg2 cells through blocking Wnt/β‐catenin and IGF‐1R/PI3K/AKT pathway. A, Immunoblot analysis was performed on normal medium (Control), CM, CM + DKK‐1 antibody, CM + DKK‐3 antibody and CM + IGFBP‐3 antibody‐treated Hepg2 cell lysate using antibodies against GAPDH, Bax and Bcl‐2. B, Hepg2 cells were co‐cultured with normal medium (Control), hAMSC‐siMOCK, hAMSC‐siDKK‐1 and hAMSC‐siDKK‐3. Western blot analysis showed that the expression level of Bcl‐2 was increased and Bax was decreased in hAMSC‐siDKK‐3 group and hAMSC‐siDKK‐1 group when compared with hAMSC‐siMOCK group. C, Quantitative analysis of the expressions of Bcl‐2 and Bax in Hepg2 cells of different groups as in (A). D, Quantitative analysis of the expressions of Bcl‐2 and Bax in Hepg2 cells of different groups as in (B). E, Hepg2 cells were co‐cultured with normal medium (Control), hAMSC‐siMOCK and hAMSC‐siIGFBP‐3. The expression levels of Bax, Bcl‐2, P‐AKT, AKT, P‐PI3K, PI3K, P‐IGF‐1R, IGF‐1R and GAPDH in Hepg2 cells of different groups were determined by Western blot analysis. F, Quantitative analysis of the expressions of P‐IGF‐1R, P‐PI3K, P‐AKT, Bcl‐2 and Bax in Hepg2 cells of different groups as in (E). G, Schematic diagram of the extracellular and intracellular mechanisms of DKK‐3, DKK‐1 and IGFBP3 effect on the apoptosis and proliferation of Hepg2 cells. DKK‐3 and DKK‐1 secreted by hAMSCs can inhibit Wnt/β‐catenin signalling by sequestering LRP5/6, triggering apoptosis and inhibiting cell growth; IGFBP‐3 secreted by hAMSCs can inhibit IGF1 signalling by sequestering IGF1, resulting in cell apoptosis

DKK‐3,^20^, ^21^ DKK‐1^22^ and IGFBP‐3^23^, ^24^ are potent inducers of apoptosis in cancer cells. Next, the effects of the hAMSC‐secreted DKK‐3, DKK‐1 and IGFBP‐3 on the apoptosis of Hepg2 cells were examined. Western blot analysis revealed that the hAMSC‐CM pre‐treated with the antibodies of DKK‐3, DKK‐1 or IGFBP‐3 significantly up‐regulated the expression of Bcl‐2 and down‐regulated the expression of Bax compared with hAMSC‐CM control group (Figure 7A,C).

DKK‐3‐, DKK‐1‐ and IGFBP‐3‐mediated Hepg2 cell apoptosis were further confirmed by the knockdown of DKK‐3, DKK‐1 and IGFBP‐3 using specific siRNAs in hAMSCs. The expression of Bax was decreased and Bcl‐2 expression was increased in Hepg2 cells when the DKK‐3, DKK‐1 and IGFBP‐3 were knockdown by siRNAs compared with hAMSC‐siMOCK group (Figure 7B,D,E,F). The hAMSCs with the knockdown of the IGFBP‐3 lost the ability to suppress IGF‐1R, PI3K and AKT activation compared with hAMSCs (Figure 7E,F). These results indicated that DKK‐3 and DKK‐1 derived from hAMSCs inhibit Hepg2 cell proliferation and promote their apoptosis through inhibiting canonical Wnt/β‐catenin signalling, and IGFBP‐3 inhibits Hepg2 cell survival through blocking IGF‐1R/PI3K/AKT signalling pathway (Figure 7G).

## 4 DISCUSSION

MSCs are pluripotent progenitor cells with the advantages of multi‐lineage differentiation, low immunogenicity and no tumorigenicity.^3^ Numerous studies have shown that the function and activity of MSCs were declined with age.^25^ It has been reported that human amniotic membrane contains MSCs‐like cells. We and others have investigated the potential therapeutic efficacy of hAMSCs in diseases.^4^, ^26^ hAMSCs have low immunogenicity, no tumorigenicity, and out of ethical debate compared to embryonic stem cells and induced pluripotent stem cells. Compared with mesenchymal stem cells from other sources, hAMSCs also have the following advantages: (a) easy to be obtained and abundant sources: the amniotic membrane could be obtained after foetal birth and will not be harmful to the donors, and about 2‐3 × 10^8^ hAMSCs can be isolated from each membrane. (b) The procedure of obtaining hAMSCs was non‐invasive, safe and out of ethical debate; (c) strong proliferation ability, easy to be expanded, multiplying one generation in 24‐36 hours.^26^ Even after expansion to 250 population doublings, hAMSCs retained long telomeres and a normal karyotype.^26^ (d) Low tissue antigenicity, high histocompatibility: hAMSCs have low expressions of the major histocompatibility class I antigen (HLA‐ABC) and no expression of the major histocompatibility class II antigen (HLA‐DR)^27^; meanwhile, it does not express HLA‐ABC co‐stimulatory molecules such as CD80, CD86 and CD40.^28^ Transplantation of hAMSCs into human beings to treat lysosomal diseases showed no obvious rejection in 50 cases of treatment.^26^ (e) High differentiation potential: hAMSCs express embryonic stem cell surface markers Oct4, SSEA‐4 and Nanog^4^, ^29^ and can give rise to adipogenic, osteogenic, myogenic, endothelial, neurogenic and hepatic lineages, inclusive of all embryonic germ layers, indicating that hAMSCs have stronger multi‐lineage differentiation potentials.^26^, ^30^ In this study, we reported that hAMSCs were multi‐potent MSCs and showed a long fusiform morphology. hAMSCs met the criteria for MSCs identification by The Association of International Cell Therapy, such as more than 95% of hAMSCs were positive for MSC markers and negative for hematopoietic stem cell markers. In addition, we also observed that there were no expressions of HLA‐DR and the co‐stimulatory molecules of HLA‐AB although there was a low expression of HLA‐ABC in hAMSCs, suggesting that the cells have a weak immunogenicity and potential immune tolerance for transplantation.

It has been reported that hAMSCs possess significant therapeutic effects in multiple disease models, including skin injury,^31^ premature ovarian insufficiency,^32^ acute liver injury,^33^ lung injury,^34^ T1 diabetes,^35^ chronic kidney disease^36^ and so on. MSCs also have shown tremendous potential in anticancer applications because of their innate ability to migrate the tumorigenic sites.^37^ However, there was no study yet to reveal the function of hAMSCs on HCC. In addition, hAMSCs have strong proliferation ability and can be expanded to 250 generation; it is easy to obtain enough number of stem cells for clinical applications. We also observed that hAMSCs have no tumorigenicity both in vivo and in vitro, suggesting that the cells might be safe for cancer therapy. Therefore, we choose hAMSCs as the therapeutic cells for cancer therapy especially for HCC therapy. To elucidate the homing and therapeutic potential of hAMSCs on HCC in mice, hAMSCs labelled with GFP were injected into the mice with HCC model via the tail vein. Whole‐body fluorescent imaging analysis showed that injected hAMSCs were homed to the sites of tumorigenesis, and some of the hAMSCs were migrated into tumour tissues, indicating that hAMSCs have the ability to home to the tumour sites in vivo. The tumour‐homing ability of hAMSCs provides the promising potential to use them as vehicles to transport therapeutic agents to the site of tumour. Next, we further demonstrated that the hAMSCs significantly inhibited the growth of tumour in mice and the possible mechanisms of the antitumour effects might be involved in their inhibiting the proliferation and promoting the apoptosis of Hepg2 cells.

It has been reported that CM derived from bone morrow MSCs,^18^ adipose MSCs^38^ and human ESCs^39^ significantly inhibited the proliferation of HCC, ovarian cancer and prostate cancer, indicating that the antitumour effect of MSCs was mediated via a paracrine signalling mechanism. Consistent with the results above, we demonstrated that hAMSCs remarkably inhibited the proliferation and increased the apoptosis of Hepg2 cells in a paracrine manner. As far as we know, this is the first time to report that hAMSCs or their deriving cytokines have the ability to inhibit Hepg2 cells proliferation and induce their apoptosis in vivo and in vitro.

To further explore the antitumour mechanisms of the cytokine‐secreted by hAMSCs, an antibody array was used to determine the potential molecules from hAMSC‐CM. Fortunately, we identified three key proteins of DKK‐3, DKK‐1 and IGFBP‐3 that were involved in the antitumour effects of hAMSCs. DKK‐3 and DKK‐1 are Wnt antagonists, which can inhibit the activation of canonical Wnt/β‐catenin signalling pathway.^40^ Wnt signalling plays an important role in regulating cell proliferation, migration and survival.^41^ It has been reported that Wnt signalling is activated in HCC, in which the activation of the signalling pathway contributes to the rapid and uncontrolled proliferation of tumour cells^42^ and promotes the epithelial‐to‐mesenchymal transition (EMT) which is involved in the metastatic and drug‐resistant phenotypes of cancer.^43^ However, activation of canonical Wnt pathway can lead to an accumulation of β‐catenin, which in turn induces cell cycle progression and promotes cell proliferation. In fact, some researchers have reported that approximately 95% HCC cases showed deregulation of the Wnt signalling cascade,^44^ 20%‐90% of HCC cases exhibit β‐catenin activation^45^, and simultaneous mutation of β‐catenin leads to 100% incidence of HCC in mice.^46^

IGFBP3 (IGF binding protein 3), as a member of the most abundant serum family of IGFBPs, is a main mediator for IGF signalling.^47^ The IGF signalling pathway is activated by binding of IGF‐1/IGF‐2 to IGF‐1R in the plasma membrane,^48^ which triggers a rapid autophosphorylation of the receptor, followed by phosphorylation of intracellular targets, ultimately leading to activation of PI3K/AKT pathway. The activation of IGF‐1R regulates several cellular processes including motility, proliferation and inhibition of apoptosis.^17^ In the serum and the extracellular fluids, IGF‐1 and IGF‐2 are usually bound to IGFBPs which regulate the IGF‐1/IGF‐1R interaction through controlling the bioavailability of IGFs.^48^ The IGFBP3 levels were very low or undetectable in human HCC samples compared with non‐neoplastic liver tissue which are 4‐ to 100‐fold higher than in HCC^49^ and IGFBP‐1 and IGFBP‐2 were also decreased in hepatoblastoma.^50^ It has been reported that the alterations in the expression of the molecules in the Wnt/β‐catenin and IGF pathways have been observed during hepatocarcinogenesis.^17^, ^40^ In the present study, we shown that hAMSCs and hAMSC‐CM markedly reduced the phosphorylation of PI3K, AKT, GSK3β and β‐catenin in Hepg2 cells, indicating that hAMSCs induced the inhibition of the proliferation and the enhancement of the apoptosis of HCC cells were involved in the inhibitions of the Wnt/β‐catenin and IGF‐1R/PI3K/AKT signalling, respectively.

Some studies reported that MSCs inhibited tumour growth through their secreted DKK‐3^51^ and DKK‐1^52^ which resulted in the down‐regulations of the expressions of the cell cycle genes through inhibiting Wnt/β‐catenin signalling. Takata et al found that DKK‐3 induced an apoptosis in ovarian cancer cells. Yulyana et al observed that the conditioned media derived from MSCs expressed high level of IGFBPs could sequester free insulin‐like growth factors to inhibit HCC cell proliferation.^18^ Lu et al also found that overexpression of IGFBP3 inhibits survival in lung cancer cells through blocking IGF1 signalling.^53^ All of these results demonstrated that Wnt/β‐catenin and IGF1 signalling have the ability to inhibit proliferation and induce apoptosis in cancer cells. Therefore, we tried to identify the exact role of Wnt/β‐catenin and IGF‐1R/PI3K/AKT signalling in hAMSCs induced the inhibition of proliferation and promotion of apoptosis in Hepg2 cells. We first used the antibody against DKK‐3, DKK‐1 and IGFBP‐3 to neutralize DKK‐3, DKK‐1 and IGFBP‐3 in the hAMSC‐CM. We found that hAMSC‐CM pre‐treated with DKK‐3 and DKK‐1 antibodies significantly inhibited the expressions of β‐catenin and PCNA in Hepg2 cells. In contrast, hAMSC‐CM pre‐treated with IGFBP‐3 antibody also decreased PCNA expression in Hepg2 cells compared with non‐treated hAMSC‐CM. These results indicated that hAMSCs inhibit Hepg2 cell proliferation through DKK‐3 and DKK‐1‐mediated the reduction of the activities of Wnt/β‐catenin signalling. Meanwhile, Western blot assay showed that the apoptosis mediated by hAMSC‐CM was reduced by the DKK‐3, DKK‐1 and IGFBP‐3 antibody neutralization, respectively, indicating that hAMSCs induce Hepg2 apoptosis through blocking both Wnt/β‐catenin and IGF‐1R/PI3K/AKT signalling. Next, the expression levels of DKK‐3, DKK‐1 and IGFBP‐3 in hAMSCs were down‐regulated by transfection with siRNA targeting DKK‐3, DKK‐1 or IGFBP‐3 mRNA to further confirm the role of DKK‐3, DKK‐1 and IGFBP‐3 in Hepg2 cells.

## 5 CONCLUSIONS

In our study, we demonstrated that hAMSCs inhibit proliferation and promote apoptosis of Hepg2 cells through blocking both Wnt/β‐catenin and IGF‐1R/PI3K/AKT signalling pathways, suggesting that administration of hAMSCs and hAMSC‐CM may be a novel strategy for treatment of HCC clinically.

## CONFLICT OF INTEREST

The authors have declared that no competing interests exist.

## AUTHORS’ CONTRIBUTIONS

Quan‐Wen Liu and Hong‐Bo Xin designed the study, collected the data, carried out data analyses and wrote the manuscript. Jing‐Yuan Li performed the experiments. Xiang‐Cheng Zhang provided the study materials. Yu Liu participated in the hAMSCs isolation and identification. Qian‐Yu Liu performed animal experiments. Ling Xiao, Wen‐Jie Zhang, Han‐You Wu and Ke‐Yu Deng contributed to the experimentation. All authors read and approved the final version of the manuscript.

## Supporting information

Table S1

Table S2

## Data Availability

The data that support the finding of this study are available from the corresponding author upon reasonable request.
