# Supplementary material for: Human amniotic mesenchymal stem cells inhibit hepatocellular carcinoma in tumour‐bearing mice
Source: J Cell Mol Med. 2020 Aug 14;24(18):10525–41. doi: 10.1111/jcmm.15668 (PMC7521292; doi:10.1111/jcmm.15668)
Supplement: Supplementary file 1 — Table S1 [file JCMM-24-10525-s001.docx]

**Table S1.**

**List of antibodies**

| **Name** | **Supplier** | **Cat no** |
| --- | --- | --- |
| CD29 | eBioscience | 11-0299-41 |
| CD90 | eBioscience | 11-0909-41 |
| CD73 | eBioscience | 12-0739-41 |
| CD105 | eBioscience | 12-1057-41 |
| CD34 | eBioscience | 12-0349-41 |
| CD45 | eBioscience | 11-0459-41 |
| HLA-ABC | eBioscience | 12-9983-41 |
| HLA-DR | eBioscience | 11-9952-41 |
| CD80 | Biolegend | 305205 |
| CD86 | Biolegend | 305405 |
| CD40 | Biolegend | 334305 |
| GAPDH | Abcam | Ab181602 |
| PCNA | Abcam | ab29 |
| Ki67 | Abcam | Ab16667 |
| Cyclin E1 | Abcam | ab133266 |
| Cyclin D1 | Abcam | ab134175 |
| Cyclin A2 | Abcam | ab181591 |
| Cyclin B1 | Abcam | ab181593 |
| Cleaved PARP | Abcam | Ab32064 |
| Cleaved Caspase-3 | CST | #9661 |
| DKK-3 | Abcam | ab186409 |
| DKK-1 | Santa Cruz | sc-374574 |
| IGFBP3 | CST | #25864 |
| β-catenin | Abcam | Ab32572 |
| Gsk3β | Abcam | Ab93926 |
| P-Gsk3β | Abcam | Ab131097 |
| IGF-1R | CST | #9750 |
| P-IGF-1R | CST | #3024 |
| PI3K | Abcam | Ab32089 |
| P-PI3K | CST | #4228 |
| AKT | Abcam | Ab8805 |
| P-AKT | Abcam | ab81283 |
| N-cadherin | Abcam | ab76011 |
| Bcl-2 | Abcam | ab182858 |
| Bax | Abcam | Ab32503 |
