# Supplementary material for: Human amniotic mesenchymal stem cells inhibit hepatocellular carcinoma in tumour‐bearing mice
Source: J Cell Mol Med. 2020 Aug 14;24(18):10525–41. doi: 10.1111/jcmm.15668 (PMC7521292; doi:10.1111/jcmm.15668)
Supplement: Supplementary file 2 — Table S2 [file JCMM-24-10525-s002.docx]

**Table S2. The cytokines with an average concentration higher than 20 pg/mL in hAMSC-CM.**

| **cytokines** | **Concentration**  **(pg/mL)** | **cytokines** | **Concentration**  **(pg/mL)** | **cytokines** | **Concentration**  **(pg/mL)** |
| --- | --- | --- | --- | --- | --- |
| **PAI-1** | 47508.89 | **IGFBP-4** | 1964.50 | **MCP-1** | 595.35 |
| **TSP-1** | 46888.60 | **MMP-2** | 1893.39 | **MCP-3** | 577.51 |
| **IGFBP-3** | 45401.20 | **RBP4** | 1864.39 | **DPPIV** | 551.44 |
| **VEGF R1** | 44504.73 | **ANG-1** | 1709.99 | **DR3** | 545.65 |
| **Dkk-3** | 37200.47 | **FAP** | 1533.35 | **IL-21** | 542.56 |
| **ANGPTL4** | 28309.84 | **ADAMTS13** | 1514.22 | **Lymphotactin** | 532.83 |
| **G-CSF** | 23869.67 | **Thyroglobulin** | 1430.57 | **APRIL** | 520.30 |
| **Periostin** | 22592.24 | **CD48** | 1401.87 | **Mer** | 453.28 |
| **Nidogen-1** | 19445.91 | **Galectin-3** | 1383.17 | **Bfgf** | 423.05 |
| **Thrombospondin-2** | 19240.45 | **Activin A** | 1357.88 | **ICAM-1** | 410.39 |
| **NCAM-1** | 16793.46 | **LRIG3** | 1321.34 | **Ck beta 8-1** | 406.86 |
| **CHI3L1** | 11930.42 | **Cystatin B** | 1310.28 | **SCF R** | 402.80 |
| **DKK-1** | 11930.42 | **IL-11** | 1297.52 | **IL-1 F8** | 399.34 |
| **TIMP-1** | 11697.53 | **Follistatin** | 1297.13 | **Syndecan-1** | 382.94 |
| **Albumin** | 11391.93 | **TFPI** | 1248.83 | **TACE** | 382.33 |
| **TIMP-2** | 9685.84 | **NSE** | 1201.45 | **CEACAM-5** | 374.70 |
| **GROa** | 9143.29 | **CTLA4** | 1143.79 | **Angiogenin** | 364.30 |
| **bIG-H3** | 9048.11 | **uPA** | 1128.00 | **Marapsin** | 357.57 |
| **B2M** | 8007.65 | **gp130** | 1057.82 | **EMMPRIN** | 347.31 |
| **AMICA** | 7198.75 | **DcR3** | 1040.19 | **Fetuin A** | 316.81 |
| **IGFBP-6** | 7002.61 | **MMP-3** | 978.35 | **Fractalkine** | 314.59 |
| **Decorin** | 6627.55 | **Cadherin-4** | 958.98 | **BMP-5** | 308.19 |
| **Ferritin** | 6498.56 | **GASP-1** | 947.92 | **IL-1 F9** | 303.67 |
| **Legumain** | 5166.15 | **MMP-10** | 930.41 | **DNAM-1** | 282.60 |
| **FLRG** | 4881.14 | **ADAM9** | 918.46 | **IL-27** | 271.78 |
| **ENA-78** | 4644.60 | **TGFb1** | 883.53 | **Galectin-9** | 269.88 |
| **sFRP-3** | 4568.18 | **FGF-7** | 865.68 | **Procalcitonin** | 266.46 |
| **MMP-1** | 4346.03 | **Cystatin C** | 855.60 | **Layilin** | 265.98 |
| **PF4** | 4101.66 | **BCAM** | 851.75 | **IL-8** | 257.09 |
| **uPAR** | 3931.90 | **Galectin-1** | 831.22 | **IL-20** | 218.19 |
| **IL-6** | 3921.84 | **Midkine** | 774.61 | **IL-31** | 217.84 |
| **Pentraxin 3** | 3614.37 | **CA125** | 729.71 | **IL-23** | 205.04 |
| **OPN** | 3568.78 | **Galectin-2** | 716.10 | **BMP-7** | 204.06 |
| **Prolactin** | 3325.15 | **Follistatin-like 1** | 711.14 | **PDGF-AA** | 199.80 |
| **Cathepsin B** | 2959.60 | **MIF** | 664.54 | **IGF-1** | 191.85 |
| **CA19-9** | 2881.31 | **Angiostatin** | 659.75 | **TNF RI** | 191.81 |
| **ANG-2** | 2515.46 | **IL-17F** | 654.16 | **IL-1 F5** | 186.32 |
| **HGF** | 2381.40 | **TLR4** | 628.21 | **hCGb** | 182.18 |
| **GRO** | 2214.43 | **CXCL16** | 611.10 | **Insulin** | 177.51 |
| **LAP(TGFb1)** | 2133.15 | **IGF-2** | 602.06 | **LRP-6** | 170.78 |
| **GCP-2** | 1984.95 | **ACE-2** | 598.81 | **CD6** | 166.76 |
| **aFGF** | 165.39 | **TNF RII** | 87.23 | **ESAM** | 43.34 |
| **Furin** | 162.42 | **Trappin-2** | 82.28 | **MIP-3b** | 39.87 |
| **IL-13 R1** | 160.93 | **IL-13** | 81.29 | **Neprilysin** | 38.31 |
| **GDF-15** | 157.32 | **4-1BB** | 81.26 | **MMP-8** | 38.13 |
| **DR6** | 152.14 | **MMP-9** | 80.21 | **BMP-4** | 37.44 |
| **Thrombomodulin** | 144.23 | **LIF** | 78.14 | **Cathepsin S** | 37.16 |
| **FGF-21** | 143.29 | **NOV** | 74.66 | **ADAM8** | 35.13 |
| **DLL1** | 141.37 | **FOLR1** | 72.12 | **Desmoglein 2** | 35.12 |
| **B7-H3** | 141.06 | **MIP-1a** | 71.92 | **HGF R** | 33.98 |
| **ICAM-2** | 138.31 | **LIMPII** | 71.56 | **BCMA** | 33.59 |
| **Cathepsin L** | 136.65 | **TSLP** | 62.52 | **IL-12p40** | 32.68 |
| **TPO** | 133.67 | **BMPR-II** | 61.60 | **Transferrin** | 32.56 |
| **CD99** | 129.50 | **Clusterin** | 59.06 | **Tie-2** | 32.18 |
| **TF** | 129.42 | **CD14** | 58.51 | **SOST** | 31.66 |
| **CRTAM** | 127.30 | **Cadherin-13** | 58.09 | **SDF-1b** | 30.92 |
| **VEGF-C** | 121.51 | **IGFBP-2** | 56.79 | **OPG** | 30.00 |
| **IL-15** | 120.75 | **Granulysin** | 56.31 | **IL-2** | 29.57 |
| **Galectin-7** | 119.52 | **CEA** | 55.89 | **IGFBP-1** | 29.10 |
| **CF XIV** | 117.76 | **Fcg RIIBC** | 55.78 | **ANG-4** | 29.06 |
| **IL-10 Ra** | 116.26 | **TREM-1** | 55.75 | **Eotaxin-3** | 27.65 |
| **TSH** | 115.23 | **HCC-4** | 55.34 | **Syndecan-4** | 25.86 |
| **IL-13 R2** | 113.78 | **NrCAM** | 52.6351.25 | **Leptin** | 25.84 |
| **Adiponectin** | 109.11 | **IL-1 RII** | 51.24 | **IL-2 Ra** | 25.48 |
| **IL-1 R3** | 106.54 | **B7-H1** | 51.24 | **IL-2 Rb** | 25.44 |
| **IGF-1R** | 104.69 | **EDA-A2** | 50.27 | **CRP** | 24.63 |
| **Testican 2** | 99.84 | **GH** | 49.81 | **Angiotensinogen** | 23.12 |
| **Adipsin** | 96.41 | **E-Cadherin** | 48.45 | **P-Cadherin** | 22.77 |
| **ANGPTL3** | 89.13 | **IL-1 F7** | 47.50 | **ADAM12** | 21.71 |
| **TIMP-4** | 88.32 | **IL-3** | 44.22 | **IL-1a** | 20.96 |
| **EGF R** | 88.07 | **CTACK** | 44.00 |  |  |
